# CT and MRI Imaging Findings of Pancreatic Mucoepidermoid Carcinoma: A Case Report and Literature Review

**DOI:** 10.2174/0115734056412112251107110519

**Published:** 2025-11-29

**Authors:** Tingting Geng, Xiufeng Li, Olena Kovalska, Zhijian Liu

**Affiliations:** 1 Department of Intensive Care Unit, Weifang People's Hospital, Weifang, Shandong, 261041, China; 2 Department of Pathology, Weifang People's Hospital, Weifang, Shandong, 261041, China; 3 Department of Radiology, Seychelles Hospital, Victoria, Seychelles; 4 Department of Radiology, Weifang People’s Hospital, Weifang, Shandong, 261041, China; 5 The First Affiliated Hospital of Shandong Second Medical University, Weifang, Shandong, 261041, China

**Keywords:** Mucoepidermoid carcinoma, Pancreas, Computed tomography, Magnetic resonance imaging, Carcinoembryonic antigen, Diffusion-weighted imaging

## Abstract

**Background:**

Although mucoepidermoid carcinoma (MEC) most commonly arises in the salivary glands, its precise etiological factors and pathogenic mechanisms remain elusive. Pancreatic involvement is an extremely uncommon manifestation, with only 15 documented cases in the medical literature to date. Owing to the absence of typical imaging features and tumor markers, the diagnosis of pancreatic MEC still relies on pathological examination.

**Case Presentation:**

This report presents the case of a 57-year-old female patient with a five-year history of abdominal discomfort. Computed tomography (CT) and magnetic resonance imaging (MRI) demonstrated a mass in the tail of the pancreas, which showed progressive ring-like delayed enhancement. The diagnosis of pancreatic mucoepidermoid carcinoma was confirmed by pathology following a laparoscopic distal pancreatectomy.

**Conclusion:**

Pancreatic MEC is exceedingly rare. In this article, the authors summarize the imaging features of this tumor and systematically review the literature to provide a better understanding of this disease.

## 1 INTRODUCTION

As a prevalent malignant neoplasm originating in the salivary glands, mucoepidermoid carcinoma (MEC) is characterized by a tri-cellular composition comprising mucous, epidermoid, and intermediate cells [1]. Among salivary gland malignancies, mucoepidermoid carcinoma (MEC) is the most prevalent histological type, accounting for approximately 30%–40%of malignant salivarygl and tumors [2, 3]. MECs are classified into three grades based on morphological and cytological features: well-differentiated, moderately differentiated, and poorly differentiated. According to the WHO classification of salivary gland tumors, MEC is recognized as a distinct diagnostic entity [4]. In contrast, under the WHO classification of pancreatic tumors, it is categorized as a variant of adenosquamous carcinoma [5]. Adenosquamous carcinoma of the pancreas is defined histologically by the presence of at least 30% malignant squamous cell components coexisting with ductal adenocarcinoma [5]. In comparison, pancreatic MEC is characterized by a mixture of mucous cells, epidermoid cells, and intermediate cells which exhibit features between basal and epidermoid cells and may form solid sheets, glandular structures, and cystic spaces. MEC is thought to arise from intermediate cells [6]. Unlike the keratinizing squamous components typical of pure squamous cell carcinoma or adenosquamous carcinoma, the epidermoid cells in MEC generally show little to no keratinization, although intercellular bridges are often visible [7]. Pancreatic MEC is an infrequently encountered histological subtype of pancreatic cancer, though its malignant potential remains unclear. This article reports the clinical features, imaging findings, and pathological characteristics of a patient with pancreatic MEC and reviews a series of documented cases within the existing medical literature. Our primary objective is to elucidate the distinctive imaging features of this rare tumor, to enhance diagnostic accuracy and inform therapeutic decision-making.

## 2 CASE PRESENTATION

A 57-year-old female patient was admitted with a history of abdominal discomfort persisting for over five years. The symptoms began following a car accident five years prior, in which she sustained rib and chest injuries. The abdominal discomfort was predominantly localized in the left upper quadrant, without radiation to the shoulder or back. She denied associated symptoms such as chest pain, chest tightness, abdominal bloating, or diarrhea. One week before admission, she presented to our hospital for further evaluation. Her medical history included hypertension for eight years, with a maximum recorded blood pressure of 210/100 mmHg. She had been treated with sustained-release nifedipine, but her blood pressure remained poorly controlled. There was no history of diabetes, cerebrovascular disease, hepatitis, tuberculosis, or other infectious diseases. Physical examination revealed a flat abdomen with localized deep tenderness in the mid-abdominal region. No rebound tenderness or palpable masses were detected. There was no hepatomegaly or splenomegaly, and Murphy’s sign was negative.

Routine laboratory findings were within normal limits: white blood cell count (WBC) 6.6×10^9^/L, red blood cell count (RBC) 4.39×10^12^/L, hemoglobin 131 g/L, and platelet count (PLT) 189×10^9^/L. Tumor marker analysis showed a mild elevation of carbohydrate antigen 19-9 (CA19-9) at 50.97 U/mL (normal range: 0.00–37.00 U/mL). Alpha-fetoprotein (AFP), carcinoembryonic antigen (CEA), and carbohydrate antigen 125 (CA-125) were all within normal ranges.

### 2.1 Imaging Findings

**Fig. (1):**
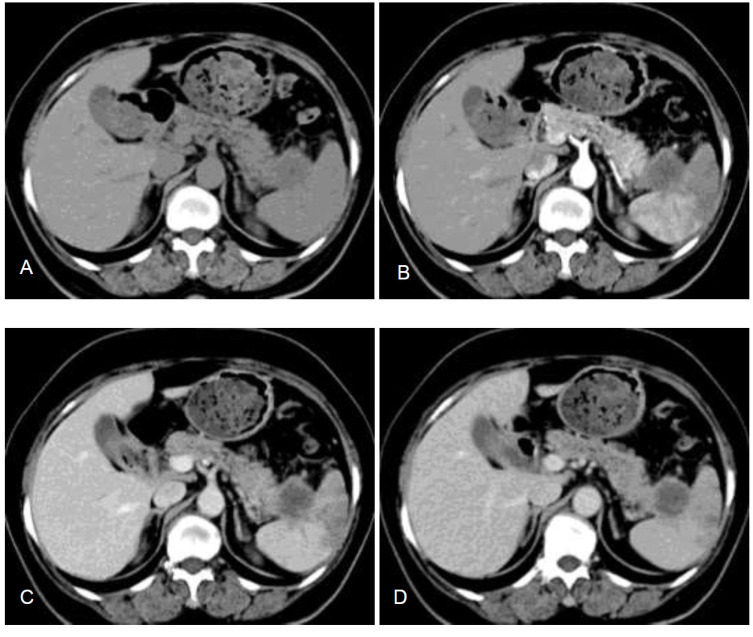
CT Imaging of Pancreatic Mucoepidermoid Carcinoma. **A:** CT scan demonstrates a round, hypodense mass in the tail of the pancreas. **B-D**: Post-contrast CT images during the arterial, portal venous, and delayed phases demonstrate peripheral ring-like delayed enhancement over time. A wedge-shaped hypodense area pointing toward the splenic hilum is seen within the spleen, suggestive of splenic infarction.

**Fig. (2):**
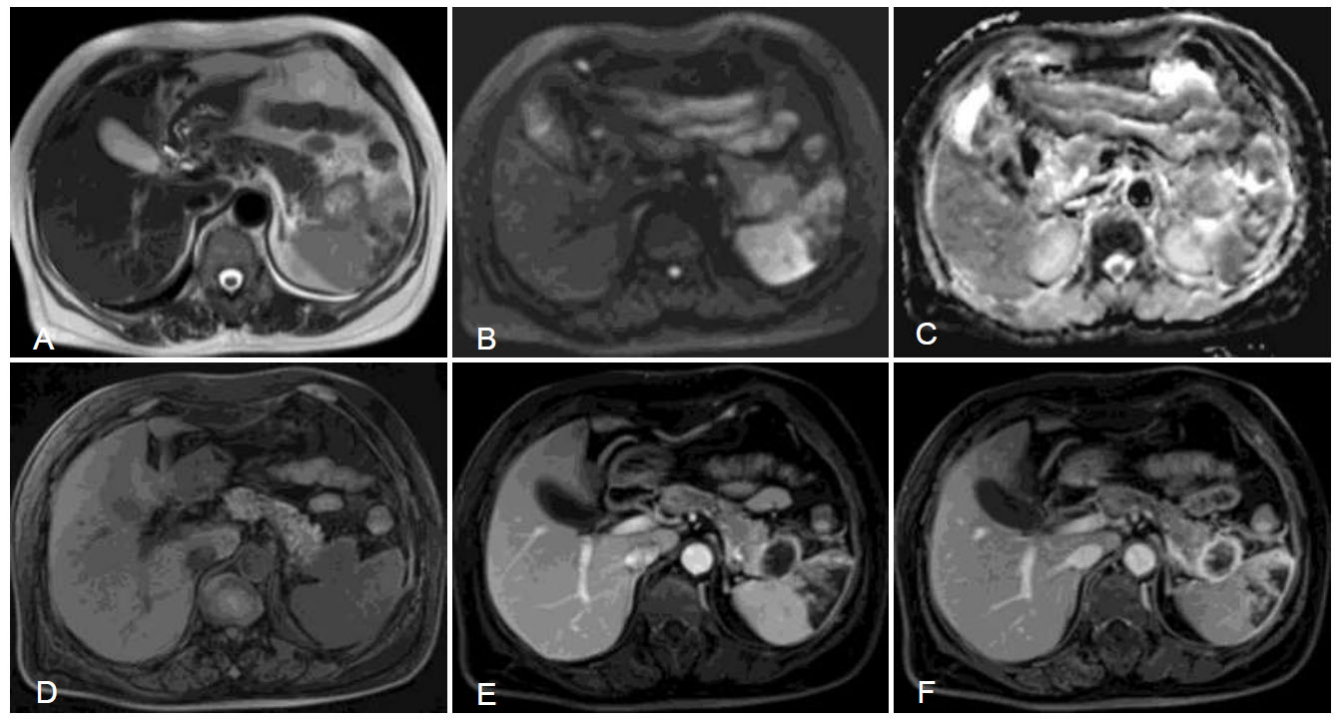
MRI Imaging of Pancreatic Mucoepidermoid Carcinoma in the Tail of the Pancreas. **A-C:** T2-weighted imaging (T2WI) shows heterogeneous hyperintensity within the lesion, including a central focus of marked hyperintensity. **D**: Diffusion-weighted imaging (DWI) demonstrates mild hyperintensity. The apparent diffusion coefficient (ADC) map reveals predominantly peripheral hypointensity with overall slightly reduced signal.**E-F:** Post-contrast imaging demonstrates peripheral ring-like delayed enhancement, with central cystic necrosis and nodular or plaque-like delayed enhancement over time. A wedge-shaped hypointensity with the apex directed toward the splenic hilum is seen within the spleen, consistent with secondary splenic infarction.

CT imaging was performed using a 64-slice spiral CT scanner (Somatom Definition, Siemens, Germany). Magnetic resonance imaging (MRI) was performed using a Siemens Magnetom Avanto 1.5T MRI scanner. The unenhanced sequences included turbo spin echo (TSE), T2-weighted imaging (T2WI), gradient echo (GRE), T1-weighted imaging (T1WI), and diffusion-weighted imaging (DWI). Dynamic contrast-enhanced imaging was performed using a 3D volumetric interpolated breath-hold examination (3D-VIBE) sequence. Computed tomography (CT) revealed a well-defined, round, hypodense mass in the tail of the pancreas, measuring approximately 2.8 cm × 3.0 cm. After contrast administration, the lesion demonstrates subtle internal enhancement with peripheral ring-like delayed enhancement over time. The mass demonstrated indistinct boundaries with the spleen. A wedge-shaped hypodense area with the apex directed toward the splenic hilum was noted within the spleen, suggestive of splenic infarction (Figs. **1A-D**). Magnetic resonance imaging (MRI) identified an ill-defined mass in the pancreatic tail. On T2-weighted imaging (T2WI), the lesion exhibited heterogeneous hyperintensity. Diffusion-weighted imaging (DWI) showed mildly increased signal intensity, and the apparent diffusion coefficient (ADC) map demonstrated predominantly peripheral hypointensity, indicating partial diffusion restriction. On T1-weighted imaging (T1WI), the lesion appeared heterogeneously hypointense. Post-contrast imaging demonstrates peripheral ring-like delayed enhancement, with central cystic necrosis and nodular or plaque-like delayed enhancement over time. The mass was observed to invade the splenic hilum, and a wedge-shaped hypodense area, again with the apex toward the splenic hilum, was visible within the spleen, consistent with secondary splenic infarction (Figs. **2A-F**).

### 2.2 Surgical and Pathological Findings

The patient underwent laparoscopic distal pancreatectomy and splenectomy. Intraoperatively, a solid mass measuring approximately 3 × 3 cm was identified in the tail of the pancreas, closely adherent to the colon. Ischemic changes were observed in the spleen. The tumor, along with the distal pancreas and spleen, was completely resected.

**Fig. (3):**
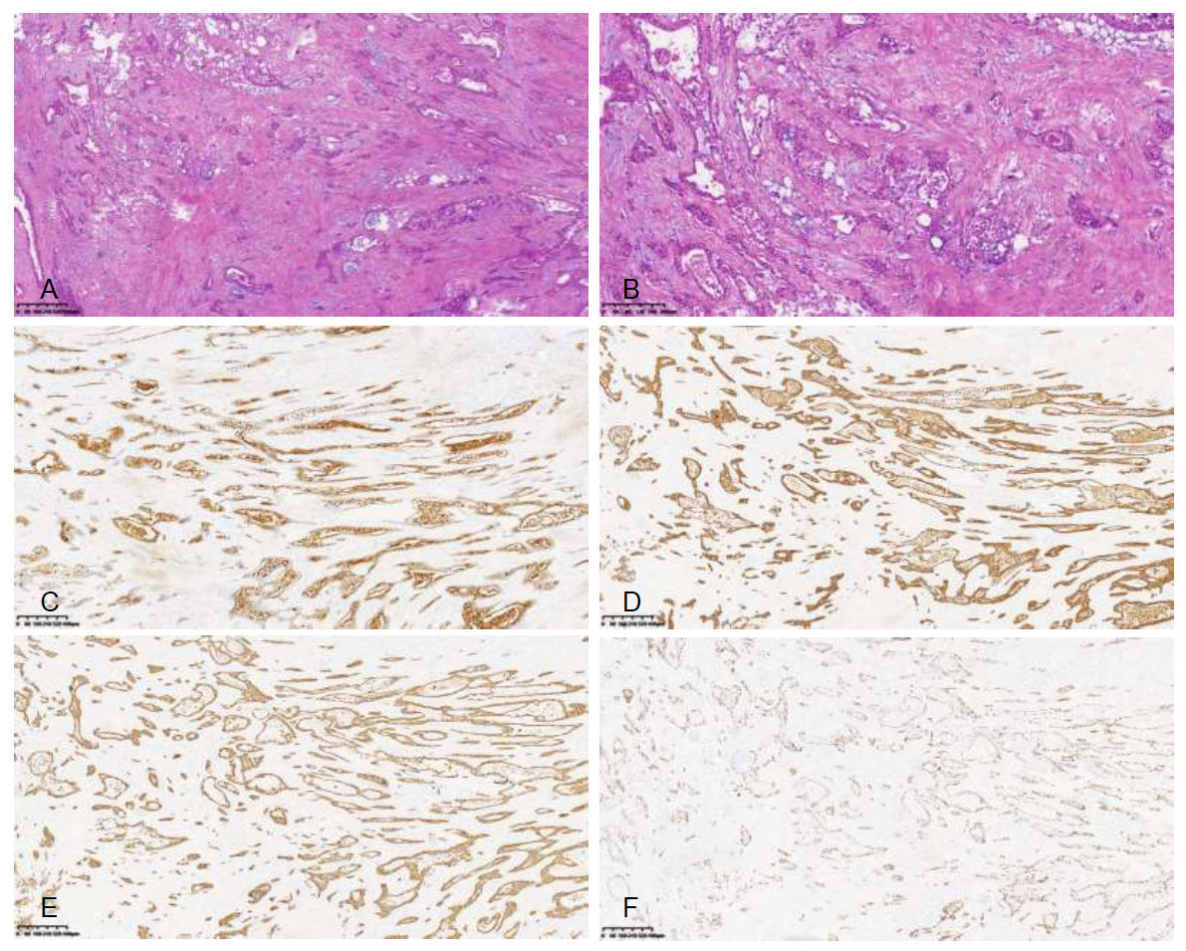
Pathological Examination and Immunohistochemical Findings. **A** (HE ×40) and **B** (HE ×100): The tumor shows sheet-like and nested growth within a hyalinized fibrous stroma. Focal keratinization is present, with occasional cells containing intracellular mucin.**C-F**: Immunohistochemical staining shows positivity for CK7 (C), CK19 (D), CK5/6 (E), and P40 (F) in the tumor cells.

Pathological examination confirmed the diagnosis of pancreatic mucoepidermoid carcinoma (MEC) located in the pancreatic tail, with a tumor size of 4 × 4 × 3 cm. The tumor was found to invade the peripancreatic adipose tissue and the splenic capsule, with evidence of vascular cancer thrombus and perineural invasion. The surgical margins of the pancreas and vascular structures were free of tumor involvement; however, lymph node metastasis was identified in one of the two lymph nodes from the splenic hilum. Immunohistochemical staining yielded the following results: CK wide (+), CK7 (+), CK19 (+), CK5/6 (+), P40 (partially +), CDX-2 (–), and CK20 (–) (Figs. **3** **A**-**F**). The patient has survived for 12 months postoperatively and remains under regular follow-up.

## 3 DISCUSSION

Pancreatic MEC is exceedingly rare [8], with only approximately 15 cases reported since its first description by Franz in 1959 [9] (Table **1**) [10]. In the present case, the tumor was composed of mucous, epidermoid, and intermediate cells, arranged in nests, solid sheets, and glandular patterns. Focal incomplete keratinization was observed among the epidermoid cells, with prominent nucleoli. Intracellular mucin was identified within gland-forming tumor cells, and stromal fibrosis was also noted.

The pathogenesis of MEC remains incompletely understood. Ohtsuki *et al.* proposed four hypotheses to explain the emergence of squamous elements in MEC: (1) differentiation of pluripotent undifferentiated cells into mucinous and/or squamous lineages, (2) origin from ectopic squamous epithelium, (3) development from metaplastic squamous cells, and (4) transformation from a pre-existing adenocarcinoma [11]. The immunohistochemical profile in this case, showing expression of CK7, CK19, CK5/6, and P40, supports the fourth hypothesis, suggesting origin from a conventional ductal adenocarcinoma with divergent differentiation.

As a rare malignancy, pancreatic mucoepidermoid carcinoma (MEC) remains poorly understood beyond its histopathological features. Clinical symptoms are generally nonspecific and may include abdominal pain, bloating, and indigestion [10]. Pancreatic MEC occurs predominantly in elderly males and exhibits considerable variation in size. This article reviews and summarizes 16 cases of pancreatic MEC (15 from the literature and the present case) (Table **1**). Among these patients, 62.5% (n=10) were male and 37.5% (n=6) female, with an age range of 48 to 75 years and a median age of 59 ± 7.9 years. Except for one case in which tumor size was not reported, the remaining tumors ranged from 2.0 to 17 cm in diameter, with a mean diameter of 6.3 ± 4.0 cm. Currently, there is no standardized treatment regimen for this disease, and curative surgery remains an effective strategy for extending survival [8]. Furthermore, postoperative multimodal therapy shows potential for improving patient prognosis [12]. Nevertheless, there is currently no evidence-based recom-mendation regarding the choice of chemotherapy regimen. In this study, five patients received palliative care or had unspecified management, three underwent pancreati-coduodenectomy, six underwent distal pancreatectomy, and two underwent total pancreatectomy. The pancreatic body and tail were the most commonly involved sites. The liver and lymph nodes were the most frequent sites of metastasis [13]. Of the 16 cases, the majority (n=11, 68.8%) involved the pancreatic body or tail, while only four were located in the pancreatic head. Thirteen cases exhibited varying degrees of local invasion or distant metastasis, with 12 (75.0%) showing metastases to the liver or lymph nodes. In addition, although classified as an indolent tumor, it frequently exhibits invasive behavior into surrounding tissues and lymph nodes, as observed in the present case. Overall prognosis is poor, and treatment modalities including surgery, radiotherapy, and chemotherapy have shown limited efficacy. Among the 16 patients, 10 died within 45 months, eight of whom died within one year of diagnosis (Table **2**).

Imaging features of pancreatic MEC are variable. Ultrasonography often reveals a poorly defined hypoechoic mass or nodule [14]. CT typically demonstrates an irregular hypodense lesion with ill-defined margins and heterogeneous density. On MRI, T2-weighted imaging (T2WI) usually shows heterogeneous hyperintensity, while T1-weighted imaging (T1WI) tends to display heterogeneous hypointensity. Cystic or necrotic changes may be present within the lesion or its central portion. Contrast-enhanced imaging shows mild to moderate heterogeneous or ring-like enhancement, with mucin-rich regions demonstrating patchy delayed enhancement. The tumor is highly invasive and prone to involve adjacent organs or metastasize distally [13]. The MRI appearance of MEC often correlates with its histological grade. Low-grade tumors typically contain abundant mucinous cells and cystic components, resulting in prominent T2 hyperintensity. In contrast, high-grade MEC usually exhibits low to intermediate signal intensity on T2WI, reflecting high cellularity and proliferative activity [15]. In the present case, the heterogeneous high signal on T2WI suggests a significant cystic or mucinous component, consistent with features often seen in lower-grade tumors.

Pancreatic mucoepidermoid carcinoma (MEC) must be differentiated from mucinous cystic neoplasm (MCN), Neuroendocrine Tumor (pNET), and pancreatic pseudocyst. MCN occurs almost exclusively in females and typically presents as a large, multilocular cystic lesion with enhancing walls or septa and is usually well-demarcated [16]. pNET shows no significant gender predilection, is highly vascular with prominent arterial-phase enhancement, and when associated with cystic degeneration, its walls demonstrate early marked enhancement [17]. Pancreatic pseudocysts often have a history of pancreatitis or trauma, exhibit smooth inner walls, and generally lack solid components.

Molecular mechanisms, particularly protein modifications and key signaling pathways, play crucial roles in oncogenesis and treatment response. In pancreatic MEC, exploring these mechanisms, such as kinase activity, post-translational modifications, and pathway aberrations, may reveal novel therapeutic targets. Furthermore, the rapid evolution of molecular biomarker research and targeted agents holds significant potential for advancing personalized treatment [18]. The integration of multi-omics technologies, including genomics, proteomics, and transcriptomics, will be essential in identifying new druggable targets and developing more effective and individualized therapeutic strategies in the future.

## 4 LIMITATION

This study is a retrospective analysis, and some data from the reviewed literature are incomplete. Additionally, pancreatic MEC is a rare disease, and the number of cases available in the literature is relatively small, which may affect the reliability of the results. Further studies with larger sample sizes are needed in the future.

## CONCLUSION

This report describes the imaging findings of a case of pancreatic mucoepidermoid carcinoma (MEC) and reviews the relevant literature. Pancreatic mucoepidermoid carcinoma (MEC) usually presents as a poorly-defined nodule or mass in the body or tail of the pancreas, often accompanied by cystic degeneration or necrosis. On contrast-enhanced imaging, it demonstrates heterogeneous or ring-like delayed enhancement. These tumors are highly aggressive, frequently invading adjacent tissues and organs or metastasizing to distant sites. When pancreatic lesions exhibit these imaging features yet do not conform to other diagnostic entities, pancreatic MEC should be considered in the differential diagnosis. However, due to the rarity of primary pancreatic MEC, its systematic imaging characteristics remain incompletely defined, and diagnosis still relies on histopathological examination. Accurate imaging assessment can contribute to tumor staging and help guide appropriate clinical treatment strategies.

## Figures and Tables

**Table 1:** Reported cases of pancreatic mucoepidermoid carcinoma in the literature. Available online under the terms of the Creative Commons Attribution Non-Commercial License 4.0. [10].

**S.no**	**Case**	**Sex/Age(year)**	**Diameter**	**Location**	**Treatment**	**Progression**	**Prognosis(month)**
1	1987	M/58	10 cm	Pancreatic tail	None	Multiple organ metastases (lung, gallbladder, spleen, adrenals,peritoneum) and lymph node metastases	2 (dead)
2	1987	F/69	T2	Pancreatic head	PD	Vessel invasion (mesocolon and superior mesenteric vein)	3(dead)
3	1991	F/48	3.5 cm	Pancreatic body	TP	Single liver metastasis	11(dead)
4	1992	M/58	3.5 cm	Pancreatic head	PD	Lymph node metastases	6 (alive)
5	1993	M/57	8.0 cm	Pancreatic body	Intraoperative radiation	Multiple organ metastases (lung, liver, colon, stomach)	2(dead)
6	1995	M/64	8.5 cm	Pancreatic tail	DP	Multiple organ metastases (liver, spleen, kidney, colon, adrenal) and peritoneal	4(dead)
7	2002	M/65	10 cm	Pancreatic body	UN	None	UN
8	2012	F/63	4.5 cm	Pancreatic body and tail	DP	None	12(dead)
9	2016	M/50	17 cm	Pancreatic head	TP	Liver, lymph node metastases	45(dead)
10	2018	M/48	4.0 cm	Pancreatic body	DP	None	23(dead)
11	2018	F/67	2.0 cm	Pancreatic body	UN	Multiple liver metastases	UN
12	2020	M/56	4.5 cm	Pancreatic tail	DP	Multiple liver metastases, multiple organ metastases	3(dead)
13	2021	M/75	8.5 cm	Not mentioned	Not available	Invasion to liver, stomach, celiac artery, portal vein and superior mesenteric vein	4(dead)
14	2024	M/51	2.8 cm	Pancreatic head	PD	Multiple liver metastases	12 (alive)
15	2024	F/65	3.0 cm	Pancreatic body	DP	Multiple liver metastases	UN
16	2024*	F/57	4.0 cm	Pancreatic tail	DP	Spleen invasion, Lymph node metastases	12(alive)

Abbreviations: F, female; M, male; PD, pancreaticoduodenectomy; DP, distal pancreatectomy; TP, total pancreatectomy; UN, unknown. *(current case)

**Table 2:** Clinical characteristics of the 16 cases of pancreatic mucoepidermoid carcinoma.

**Variable**	**Number (%) or Median(IQR)**
Age Median(range)	59 ± 7.9 (48~75)y
**Sex**	
Male	10(62.5%)
Female	6(37.5%)
**Location**	
Head	4(25%)
Body or tail	11(68.75%)
**Diameter**	6.3 ± 4.0 (2.0~17.0)cm
**Treatment**	
PD(Pancreaticoduodenectomy)	3(18.75%)
DP(Distal pancreatectomy)	6(37.5%)
TP(Total pancreatectomy)	2(12.5%)
Palliative care or unknown	5(31.25%)
**Metastasis or invasion**	13(81.25%)
**Outcome**	
Alive	3(18.75)
Death	10(62.5)
Unknown	3(18.75)

## Data Availability

All data generated or analysed during this study are included in this published article.

## LIST OF ABBREVIATIONS

MEC: Mucoepidermoid carcinoma
CT: Computed Tomography
MRI: Magnetic Resonance Imaging
AFP: Alpha-Fetoprotein
CEA: Carcinoembryonic Antigen
CA-125: Carbohydrate Antigen 125

## STANDARDS OF REPORTING

CARE guidelines were followed.
